# Sphingolipid Metabolism Dysregulation Drives Immune Microenvironment Remodeling and Predicts Prognosis in Bladder Cancer

**DOI:** 10.1155/ijog/6085216

**Published:** 2025-11-02

**Authors:** Zechun Peng, Jie Yang, Ruipeng Jia, Tianshi Wu, Songyun Zhao

**Affiliations:** ^1^ Department of Urology, The Second Affiliated Hospital of Hainan Medical University, Haikou, China, hainmc.edu.cn; ^2^ Department of Urology, Nanjing First Hospital, Nanjing Medical University, Nanjing, China, njmu.edu.cn

**Keywords:** bladder cancer, immune microenvironment, personalized therapy, prognostic model, sphingolipid metabolism

## Abstract

The role of sphingolipid metabolism (SM) dysregulation in promoting bladder cancer (BLCA) progression and influencing patient prognosis has been well documented. To enhance therapeutic strategies, we aimed to identify key sphingolipid metabolism–related genes (SMGs) and develop a prognostic signature for personalized BLCA management. In this study, 430 BLCA samples from The Cancer Genome Atlas (TCGA) were analyzed via univariate Cox regression to screen critical SMGs involved in tumor progression. A LASSO regression model was applied to minimize overfitting, followed by multivariable Cox regression to construct and validate a SMG‐based prognostic signature in an independent cohort. Key findings revealed that SM dysregulation correlated with poor clinical outcomes and eight pivotal prognostic genes (ATP13A2, PCSK2, NR2F1, GSDMB, NFASC, NTF3, LGALS4, and SREBF1) were identified. The resulting risk model demonstrated robust prognostic performance with AUC values of 0.772 (training cohort) and 0.725 (validation cohort). Notably, high‐risk patients exhibited a highly active immunological profile characterized by elevated immune scores and enhanced functionality across 26 immune components, including increased infiltration of NK cells, CD8^+^ T cells, and elevated cytolytic activity. These results suggest that SM dysregulation may drive immunomodulatory changes in BLCA microenvironments, offering mechanistic insights into tumor immune evasion. This study provides a novel biomarker tool for risk stratification and highlights SM pathways as potential therapeutic targets for BLCA patients with immune microenvironment dysregulation.

## 1. Introduction

Bladder cancer (BLCA) is one of the most prevalent malignancies globally, with approximately 550,000 new cases and 200,000 deaths recorded in 2018 [1]. Urothelial carcinoma constitutes the primary histological subtype of BLCA, which is clinically categorized into nonmuscle‐invasive bladder cancer (NMIBC) or muscle‐invasive bladder cancer (MIBC) [2], based on their distinct treatment regimens and prognostic implications. In the United States, prominent risk factors for BLCA include cigarette smoking and occupational exposure to aromatic amines, which also elevate the recurrence risk [3]. For superficial bladder tumors, transurethral resection of the bladder tumor (TURBT) is the standard surgical intervention, often combined with intravesical therapy regardless of concomitant treatment strategies [3, 4]. Radical cystectomy (RC) remains the cornerstone treatment for MIBC patients [4]. The extent of bladder wall invasion is intimately associated with clinical outcomes, including treatment selection and disease progression [5]. Approximately 75% of patients present with NMIBC at diagnosis, while only ~10% are initially diagnosed with MIBC or metastatic disease [6]. The economic burden of BLCA management, encompassing treatment costs, surveillance requirements, and adverse effect mitigation, remains substantial [7]. Despite advancements in early detection and multimodal therapies, recurrence and metastatic progression remain significant clinical challenges. Therefore, identifying robust early diagnostic and prognostic biomarkers for BLCA progression is critical, alongside the development of innovative detection techniques and therapeutic strategies to further improve patient outcomes.

The progression of malignant tumors is closely associated with the tumor immune microenvironment (TIME), a key component of the tumor microenvironment (TME) defined by immune cell infiltration [8]. Immune cells critically influence tumor progression through the secretion of cytokines and growth factors that modulate neighboring cell behavior, thereby regulating tumor survival and proliferation [9]. In BLCA, the nontumor constituents of the microenvironment—dominated by tumor‐infiltrating immune cells—are strongly correlated with patient prognosis [10]. This underscores the essential role of TIME in tumor development, metastasis, and pathophysiological mechanisms specific to BLCA [11]. Systematic evaluation of TIME immunophenotypes provides critical insights for advancing immunotherapeutic strategies and improving clinical outcomes in BLCA patients.

Sphingolipids, a class of bioactive lipids, regulate cell signaling and modulate cell apoptosis and proliferation in cancer [12]. Dysregulation of sphingolipid metabolism (SM) contributes to tumorigenesis and represents a promising therapeutic target [13]. Specific sphingolipid metabolites, such as sphingosine‐1‐phosphate (S1P) and ceramides, function critically in governing cell death mechanisms in oncogenesis [14]. Advances in molecular and pharmacological techniques have enhanced our understanding of SM signaling pathways and their therapeutic potential in cancer treatment [12]. While research has extensively investigated SM′s role in various malignancies, particularly in developing targeted therapies, the precise function of sphingolipid metabolism–related genes (SMGs) in BLCA remains elusive.

In this study, we performed a comprehensive analysis of SMGs to elucidate their roles in influencing survival outcomes and disease progression in BLCA patients. Additionally, we constructed a BLCA‐specific prognostic risk model based on SMG expression profiles. Our analysis demonstrated that SMGs are significantly associated with TME and TIME, highlighting their predictive and mechanistic relevance in BLCA progression. These findings provide a framework for exploring the molecular mechanisms underlying BLCA pathogenesis, potentially informing the design of targeted therapeutic strategies and personalized clinical management approaches for BLCA patients.

## 2. Materials and Methods

### 2.1. Data Analysis

RNA‐Seq data for BLCA and corresponding clinical data were obtained from the UCSC Xena database, comprising 19 normal samples and 411 BLCA samples. Initially in log2(FPKM) format, the data were converted to FPKM, then to TPM, and finally to log2(TPM + 1) format (TCGA‐BLCA). Additional BLCA RNA‐Seq data from the GEO database (GSE236932 and GSE195768, totaling 85 samples) underwent similar transformation and batch effect correction for validation.

### 2.2. Identification of SMGs

SMGs were extracted from the GeneCards database (http://genecards.org/) using a relevance score threshold of > 4, yielding 1231 genes for subsequent analysis.

### 2.3. Differential Expression Analysis of SMGs

The “limma” R package and Wilcoxon rank‐sum test were employed to identify differentially expressed SMGs (DEGs) between normal bladder tissue and BLCA tissue, with criteria of |log2foldchange| > 1 and *q* < 0.05 (FDR adjusted).

### 2.4. Construction of a Prognostic SMG Model

BLCA patients were randomly divided into training and testing cohorts. Univariate Cox regression (UniCox) identified SMGs significantly associated with overall survival (OS). LASSO Cox regression (“glmnet” package) with tenfold cross‐validation selected genes for the prognostic model. Risk scores were calculated as $\begin{array}{c} \text{BLCA}\;\text{risk}\;\text{score}=\overset{n}{\underset{i=1}{\sum }}\beta_{i}\times E_{i} \end{array}$

where *β*_i_ is the regression coefficient and *E*_i_ is the expression level of SMG *i*. Patients were stratified into high‐ and low‐risk groups based on the median risk score. Model performance was validated using Kaplan–Meier (KM) survival analysis, log‐rank tests, receiver operating characteristic (ROC) curves (“survivalROC” package), and an external GEO dataset.

### 2.5. Independent Prognostic Analysis

The prognostic value of the BLCA risk score was compared with clinical factors (gender, age, stage, and TNM status) using UniCox and multivariate Cox (MultCox) regression.

### 2.6. GO and KEGG Enrichment Analysis

DEGs between high‐ and low‐risk groups were analyzed for KEGG and GO pathway enrichment (“clusterProfiler” package), with significance set at FDR‐corrected *p* < 0.05. Results were visualized using “enrichplot” and “ggplot2.”

### 2.7. Gene Set Variation Analysis (GSVA)

Biological pathway differences between risk groups were assessed using GSVA with MSIGDB gene sets (“c2.cp.kegg.v7.1.symbols” and “c5.go.v7.5.1.symbols”) (http://gsea-msigdb.org/gsea/msigdb).

### 2.8. Gene Set Enrichment Analysis (GSEA)

GSEA was performed using the same MSIGDB gene sets to identify enriched pathways in high‐ versus low‐risk groups.

### 2.9. Immune Microenvironment Characterization

Single‐sample gene set enrichment analysis (ssGSEA) quantified 29 immune features per sample. Estimation, stromal, and immune scores, along with tumor purity, were compared between risk groups. Correlations between immune function and prognostic SMGs were evaluated.

### 2.10. Single‐Cell Data Analysis

For single‐cell data analysis, Dataset GSE222315 from GEO, including 13 samples of BLCA and adjacent normal tissues (nine cancer patients and four control samples), was performed using R′s Seurat package, involving various stages of analysis, including data quality control, data standardization, and normalization of the count matrix, which was performed using the “NormalizeData” function [15, 16]. Data filtering steps included removing genes detected in fewer than 200 cells and excluding cells with nFeature_RNA (number of genes per cell) ≥ 7500, nCount_RNA (total UMIs) ≥ 50,000, or mitochondrial gene content ≥ 20%. Following normalization and PCA dimensionality reduction, unsupervised clustering was conducted using Seurat′s FindNeighbors and FindClusters functions. Clustering was performed with a resolution of 0.1 using the UMAP algorithm, after which cell subpopulations were annotated. A risk score (RiskScore) was calculated in the single‐cell dataset based on model‐included genes and their corresponding coefficients, and its expression distribution across different cell populations was analyzed.

### 2.11. Immunohistochemical Validation

Immunohistochemistry data for key model genes (PCSK2, NFASC, NTF3, NR2F1, and GSDMB) in normal and BLCA tissues were obtained from the Human Protein Atlas (HPA; http://proteinatlas.org/).

### 2.12. In Vitro Experiments

#### 2.12.1. Cell Transfection

TCCSUP cells were maintained in MEM/NEAA with 10% FBS and 1% penicillin–streptomycin and transfected using Lipofectamine 2000 in 6‐well plates.

#### 2.12.2. qRT‐PCR

Total RNA from TCCUSP cells was extracted with TRIzol, reverse‐transcribed (TransScript First‐Strand cDNA Synthesis Kit, TaKaRa kit), and amplified via SYBR Green PCR (SYBR Green MasterMix, TaKaRa, Japan). The following primer sequences were used: F‐ACTGGACACGAAACAGCAGAT and R‐CTGCGGAAGTCAATCACCAGG.

#### 2.12.3. Cell Viability Assay

Cells were stained using the Yeason EDU kit and DAPI, and their viability was analyzed based on the EDU/DAPI ratio.

#### 2.12.4. Apoptosis

Analysis of apoptotic cells was performed using an Annexin V‐FITC/PI binding assay followed by flow cytometry.

#### 2.12.5. Wound Healing Assay

TCCSUP cells were seeded in a 6‐well plate, with half the wells transfected with si‐NFASC. Once confluent, a standardized scratch was created in the monolayer using a sterile micropipette tip. The medium was replaced with serum‐free MEM to minimize mitotic effects. Images were captured under a phase‐contrast microscope (Olympus IX71, Japan) at 0, 24, and 48 h postscratch to quantify cell migration. Wound closure was analyzed using ImageJ software to calculate the remaining wound area. Cell migratory capacity was evaluated by the wound closure percentage at 24 and 48 h, defined as follows: [(initial wound area − remaining area)/initial wound area] × 100*%*.

#### 2.12.6. Transwell Assay

Treated cells (4 × 10^4^) suspended in serum‐free medium were seeded into Matrigel‐coated upper chambers of a Transwell system. Lower chambers were filled with 10% FBS medium to serve as a chemoattractant. After a 24‐h incubation, cells that invaded through the membrane were fixed with ice‐cold formaldehyde, stained with crystal violet, and quantified under an inverted Olympus IX71 microscope (Japan). Five random fields per well were imaged and counted to assess migratory capacity.

#### 2.12.7. Colony Formation

TCCSUP cells were cultured for 7 days before being seeded at a density of 500 cells/well in 6‐well plates. Following incubation, cells were fixed with 4% paraformaldehyde for 20 min and stained with 4% crystal violet, and colonies were counted under a light microscope.

### 2.13. Statistical Analysis

Prior to intergroup comparisons, normality tests were performed on the data. If the data conformed to a normal distribution, Student′s *t*‐test was used; otherwise, the Wilcoxon rank‐sum test was applied. KM survival analysis with log‐rank testing was applied to evaluate survival differences between low BLCA and high BLCA patient groups. Multivariable Cox proportional hazards regression was conducted to identify independent prognostic factors for OS in BLCA. ROC curves were generated to assess the predictive accuracy of the BLCA risk score model. All analyses were executed in R Version 4.2.0, with statistical significance defined at *p* < 0.05. Asterisks denote significance levels:  ^∗^ *p* < 0.05,  ^∗∗^ *p* < 0.01, and  ^∗∗∗^ *p* < 0.001.

## 3. Results

### 3.1. Analysis of SMGs Between Normal Tissue and BLCA

The workflow is illustrated in Figure 1a. Differential gene expression (DGE) analysis identified 133 SMGs with decreased expression and 48 SMGs with increased expression in BLCA patients compared to normal tissues. These DEGs were subsequently used for prognostic analysis, as depicted in Figure 1b,c.

Figure 1Identification and analysis of sphingolipid metabolism–related genes (SMGs) in bladder cancer (BLCA). (a) Workflow diagram illustrating the process of identifying differentially expressed SMGs and constructing a prognostic risk model. (b) Heatmap depicting the expression profiles of SMGs in BLCA and normal samples. (c) Volcano plot showing the differential expression of SMGs between BLCA and normal samples.

**Figure:**
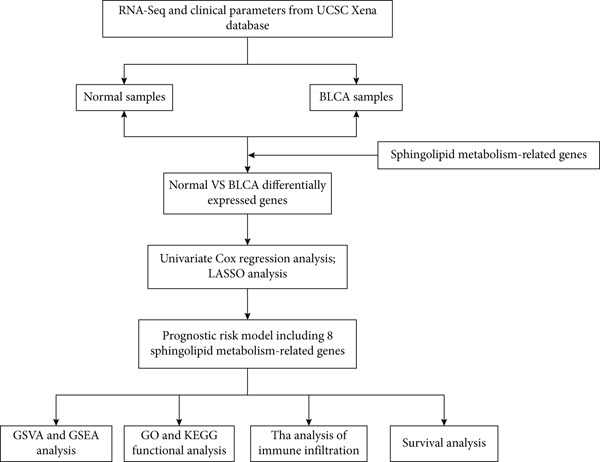
(a)

**Figure:**
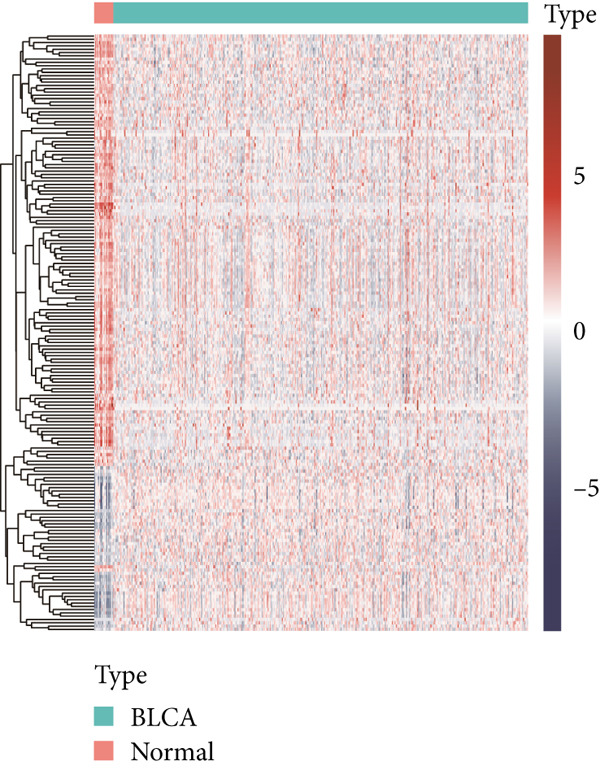
(b)

**Figure:**
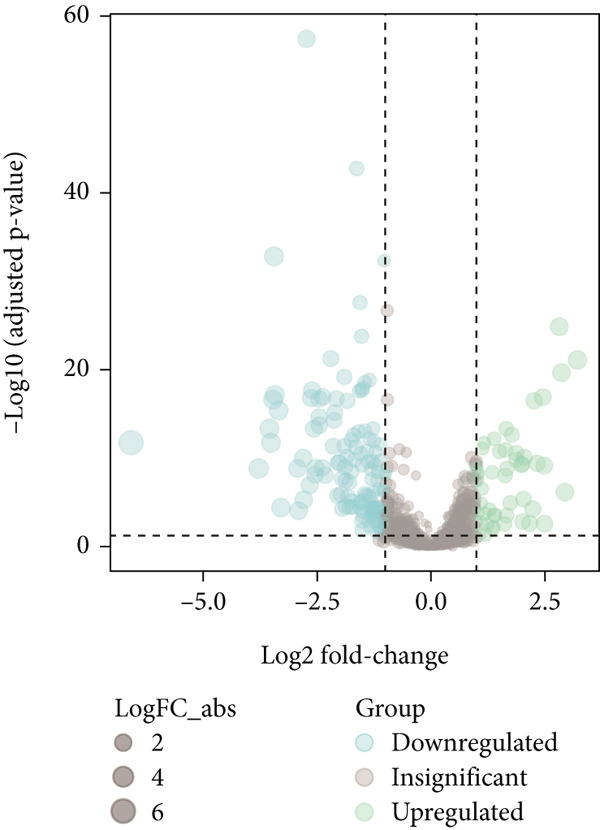
(c)

### 3.2. Prognostic BLCA Risk Score Model Established in Training Cohort

UniCox regression analysis was performed on 8133 SMGs, revealing 54 SMGs significantly associated with prognosis (Figure 2a). Further refinement using LASSO regression analysis reduced the number of candidate SMGs (Figure 2b,c). The selection was made by assessing clinical relevance and biological function, and further LASSO regression analysis identified five key SMGs (PCSK2, NFASC, NTF3, NR2F1, and GSDMB) strongly linked to prognosis (Figure 2d). A predictive BLCA risk score model was developed based on these eight SMGs, and the risk score for each patient was calculated using the formula described in the Materials and Methods section. Patients were then stratified into low BLCA and high BLCA risk groups based on their scores.

Figure 2Establishment of prognostic risk score model. (a) Forest plot illustrating the hazard ratios (HRs) and 95% confidence intervals (CIs) for 54 SMGs in BLCA patients. (b) Partial likelihood deviance plot as a function of the log(*λ*) value during the LASSO regression model training. (c) Coefficient profiles of the genes across a range of *λ* values in the LASSO regression. (d) HR forest plot for the final set of eight genes selected by the LASSO regression model.

**Figure:**
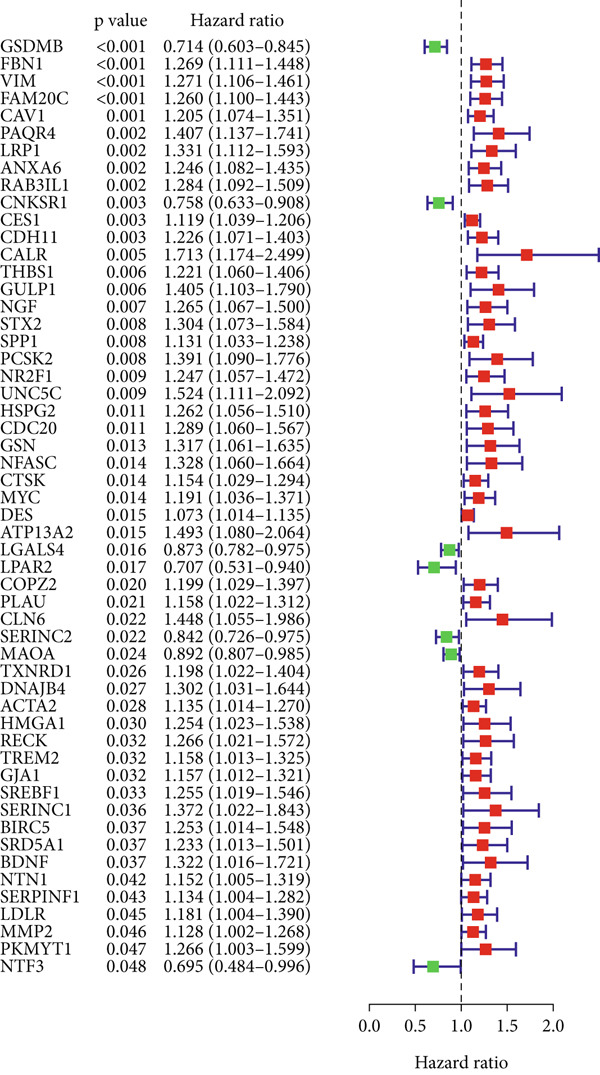
(a)

**Figure:**
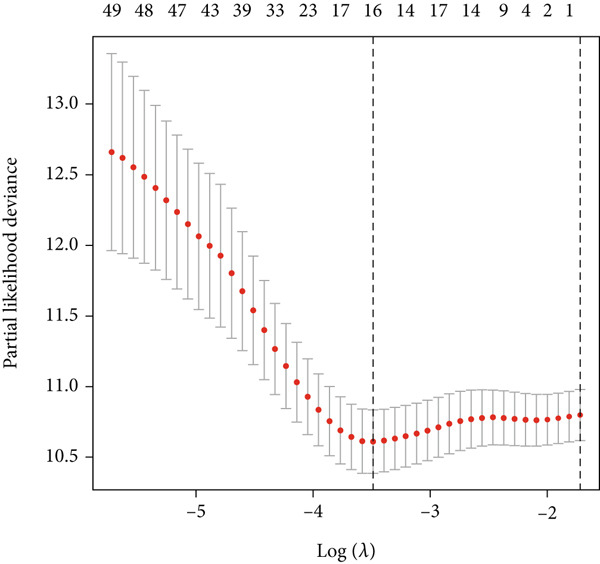
(b)

**Figure:**
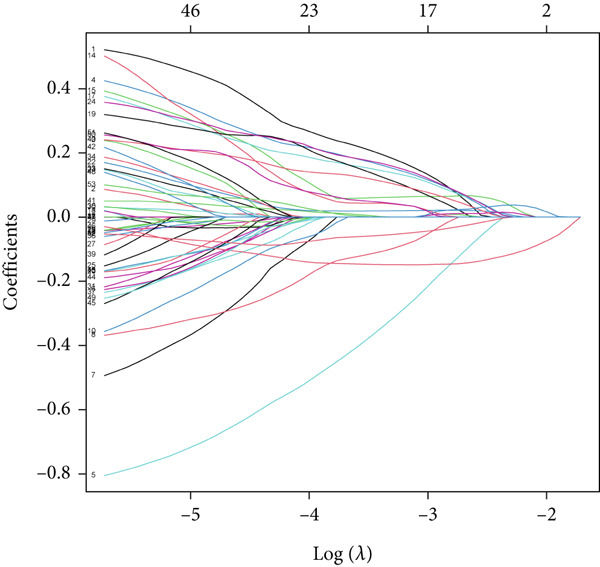
(c)

**Figure:**
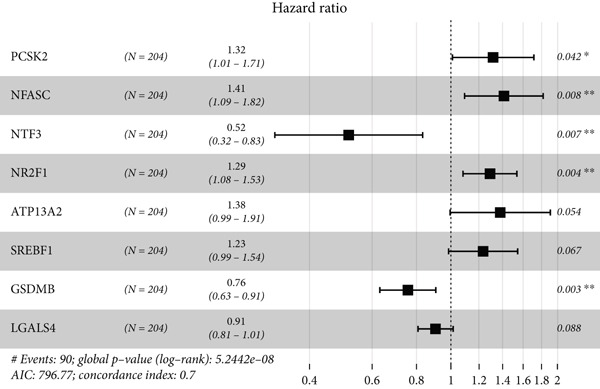
(d)

### 3.3. Correlation Between BLCA Risk Score and Clinical Parameters

Using the median risk score as the cutoff, patients in the training cohort were classified into low BLCA (*n* = 102) and high BLCA (*n* = 102) groups (Figure 3a,b). The high BLCA group exhibited significantly poorer prognosis (Figure 3c,d). To validate the model, the same cutoff was applied to the validation cohort, dividing patients into low BLCA (*n* = 98) and high BLCA (*n* = 104) groups (Figure 3e,f). Consistently, high BLCA patients in the validation cohort showed worse OS, confirming the model′s predictive accuracy (Figure 3b,f).

Figure 3Risk score analysis and survival outcomes in BLCA patient cohorts. (a, b) Dynamic changes in risk scores over time for high‐risk (red) and low‐risk (blue) groups in the training or testing cohort. (c, d) Kaplan–Meier survival curves comparing overall survival between high‐risk and low‐risk groups in the training or testing cohort. (e, f) Distribution of survival times for patients classified as dead (red) or alive (blue) based on their risk scores in the training or testing cohort. (g, h) Heatmap of prognostic model genes in the training or testing cohort.

**Figure:**
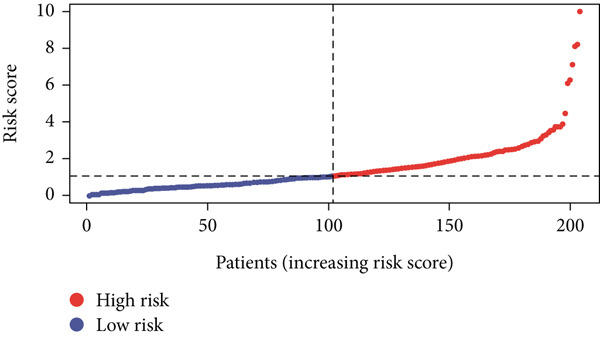
(a)

**Figure:**
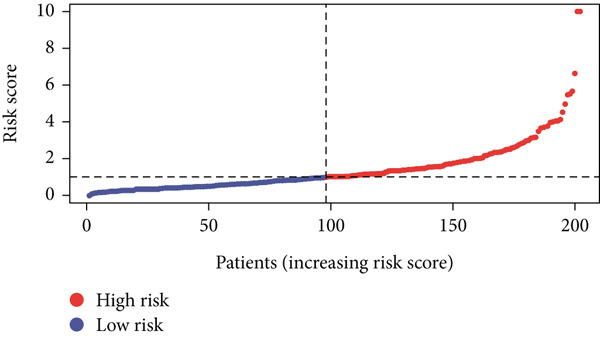
(b)

**Figure:**
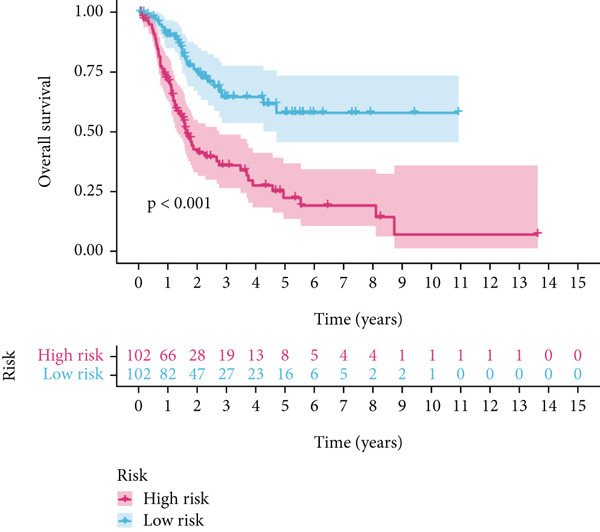
(c)

**Figure:**
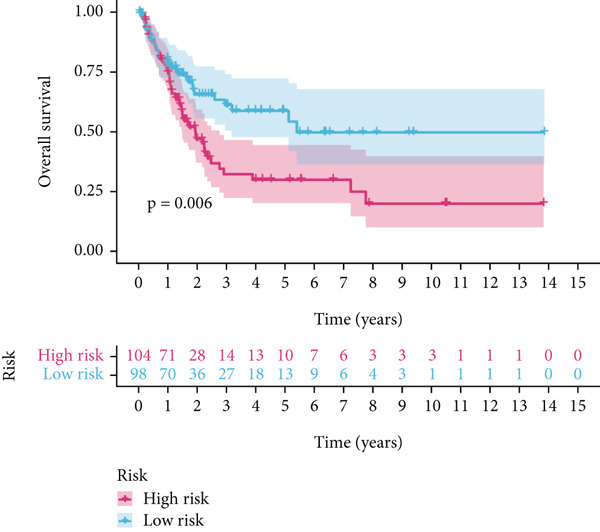
(d)

**Figure:**
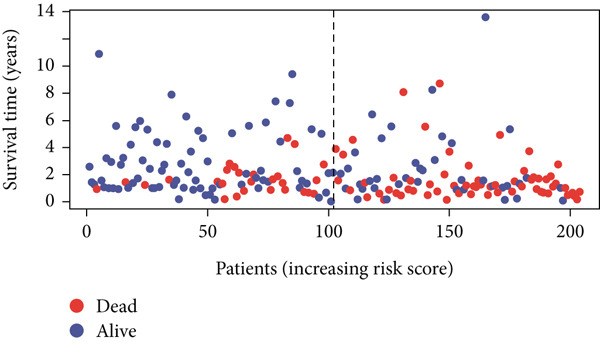
(e)

**Figure:**
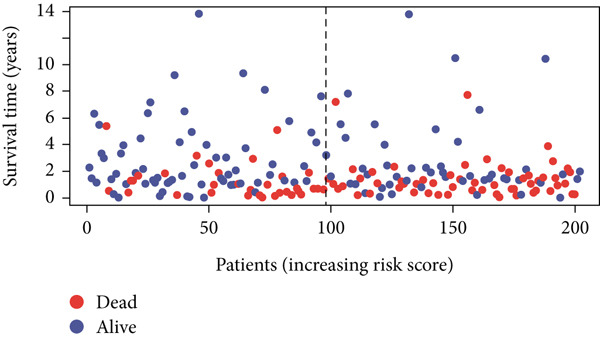
(f)

**Figure:**
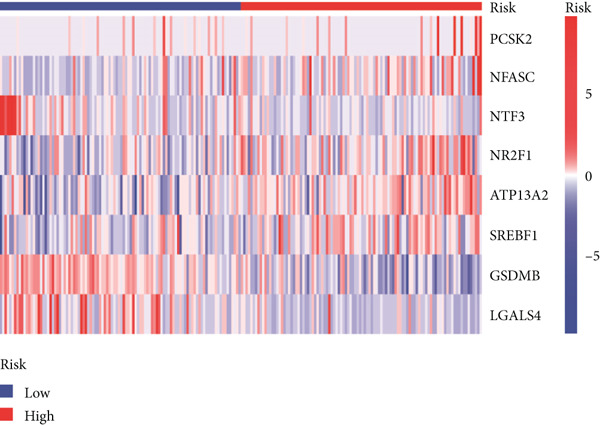
(g)

**Figure:**
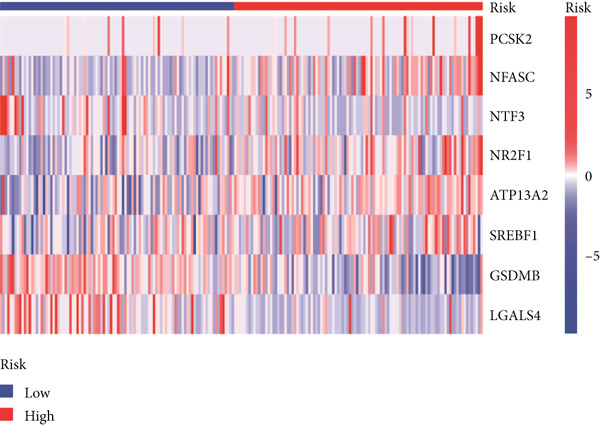
(h)

UniCox analysis demonstrated that both the BLCA risk score and tumor stage were significantly associated with OS (Tables 1 and 2). MultCox analysis further confirmed that the BLCA risk score and stage were independent prognostic factors (Tables 3 and 4). ROC curves for 1‐, 3‐, and 5‐year survival yielded area under the curve (AUC) values of 0.772 (training cohort) and 0.725 (validation cohort), indicating superior predictive performance of the BLCA risk score compared to other clinical parameters (Figures 4a, 4b, 4c, and 4d). External validation using a GEO dataset corroborated these findings.

**Table 1:** Univariate analysis revealed that risk score and stage were associated with overall survival in the training cohort.

	**HR**	**HR.95L**	**HR.95H**	**p** **value**
Age	1.029091	1.005441	1.053297	0.015632
Gender	0.891552	0.556761	1.42766	0.632754
Stage	2.258404	1.661269	3.070177	2.00e−07
T	2.029222	1.467739	2.805501	1.85e−05
N	1.987654	1.345678	2.876543	2.10e−05
M	2.112345	1.567890	2.945612	1.65e−05
RiskScore	1.431055	1.310374	1.562849	1.54e−15

**Table 2:** Univariate analysis revealed that risk score and stage were associated with overall survival in the testing cohort.

	**HR**	**HR.95L**	**HR.95H**	**p** **value**
Age	1.03415	1.011455	1.057354	0.003017
Gender	0.746389	0.465782	1.196046	0.224045
Stage	1.496159	1.132301	1.976939	0.004598
T	1.523142	1.120893	2.069745	0.007158
N	1.635472	1.193562	2.134678	0.006543
M	1.482391	1.095874	2.056723	0.007632
RiskScore	1.061585	1.013771	1.157318	0.044904

Figure 4ROC curves for predictive risk models at different time points and for various factors. (a, c) ROC curves illustrating the predictive performance of the risk model at 1, 3, and 5 years in the training or testing cohort. (b, d) Comparative ROC curves for different clinical factors in the training or testing cohort.

**Figure:**
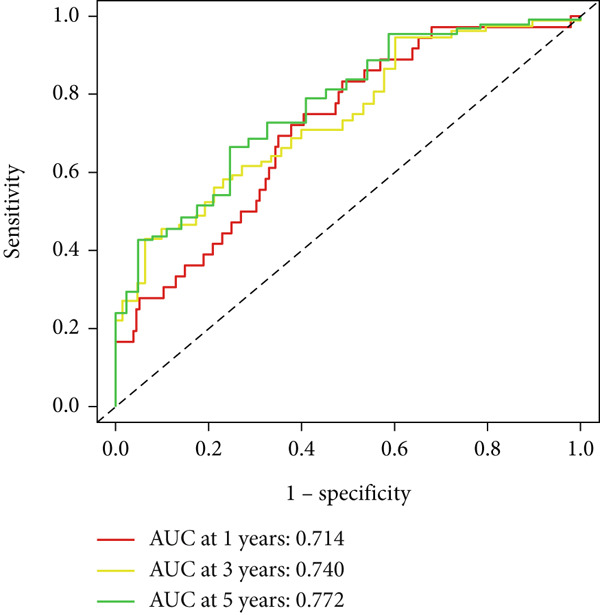
(a)

**Figure:**
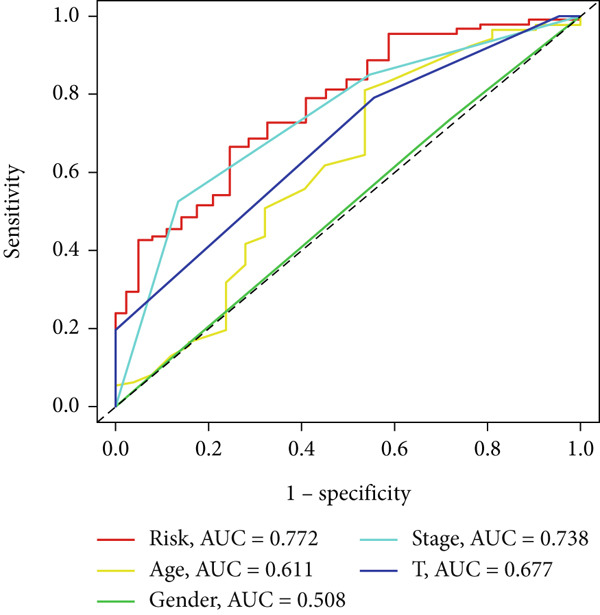
(b)

**Figure:**
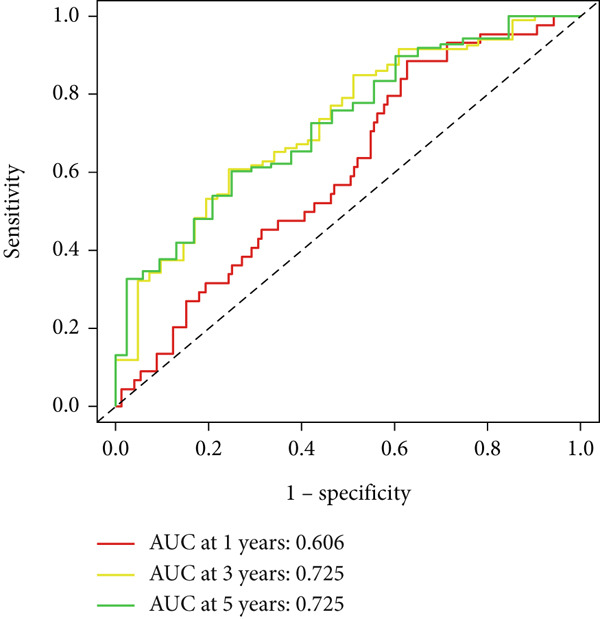
(c)

**Figure:**
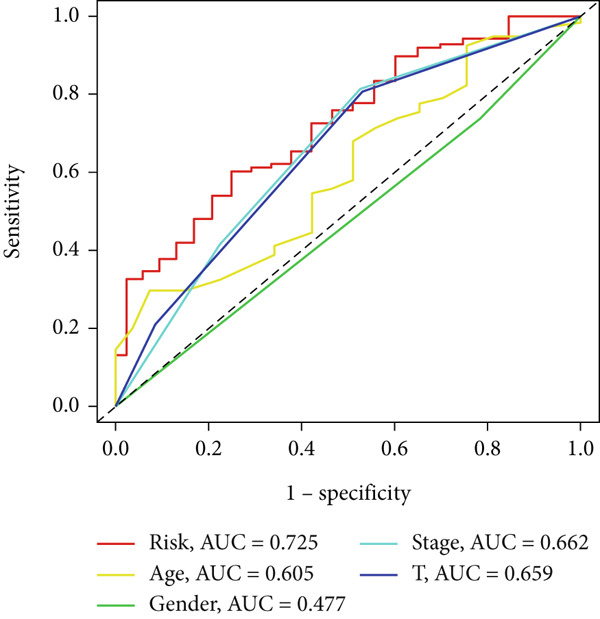
(d)

**Table 3:** Multivariate analysis revealed that risk score and stage were associated with overall survival in the training cohort.

	**HR**	**HR.95L**	**HR.95H**	**p** **value**
Age	1.024814	0.999116	1.051173	0.058533
Gender	1.072786	0.646573	1.779953	0.785646
Stage	1.823717	1.279749	2.598904	0.000885
T	1.458481	0.981047	2.168261	0.062127
N	1.632579	1.102345	2.413678	0.055873
M	1.547893	0.973456	2.214567	0.064981
RiskScore	1.381363	1.256343	1.518824	2.48e−11

**Table 4:** Multivariate analysis revealed that risk score and stage were associated with overall survival in the testing cohort.

	**HR**	**HR.95L**	**HR.95H**	**p** **value**
Age	1.033135	1.010562	1.056212	0.003826
Gender	0.716028	0.443917	1.154937	0.170865
Stage	1.236681	0.851641	1.795803	0.264346
T	1.411015	0.921291	2.161058	0.113412
N	1.372486	0.899213	2.119547	0.120345
M	1.426789	0.945678	2.175432	0.115678
RiskScore	1.065878	0.963667	1.17893	0.014826

Stratified survival analysis revealed significantly worse prognosis for high‐risk patients (Figure 5a,b). The AUC values for 1‐, 3‐, and 5‐year ROC curves were 0.802, 0.785, and 0.832, respectively, underscoring the model′s reliability (Figure 5c). The model also outperformed traditional clinical parameters in prognostic accuracy (Figure 5d).

Figure 5Survival analysis by BLCA prognostic risk model in GEO database. (a) Kaplan–Meier survival curves comparing overall survival between high‐risk (red) and low‐risk (blue) groups. (b) Distribution of survival times for patients ordered by increasing risk score (up) and risk score stratification for high‐risk and low‐risk groups (bottom). (c) ROC curves for predicting survival at 1, 3, and 5 years. (d) Comparative ROC curves for different clinical factors.

**Figure:**
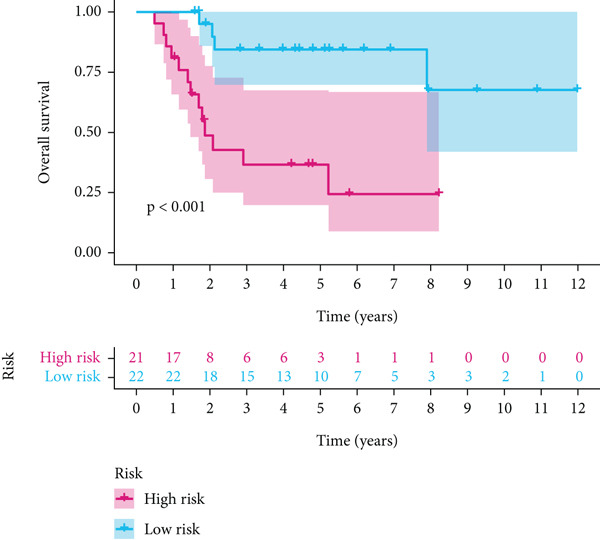
(a)

**Figure:**
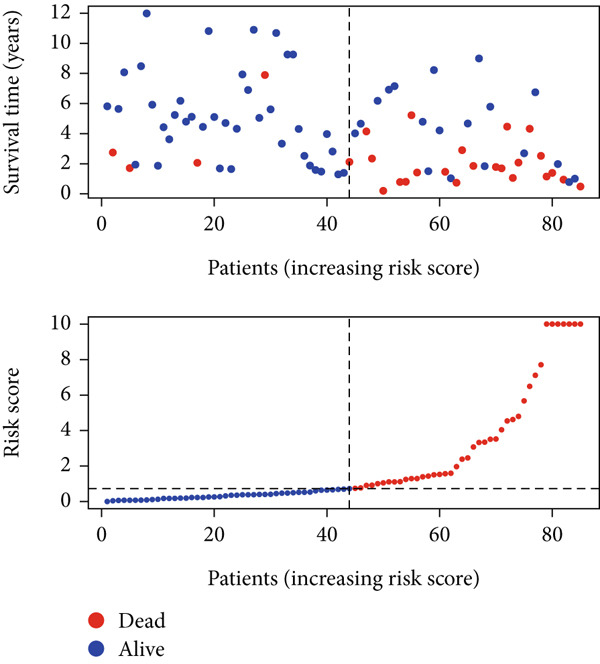
(b)

**Figure:**
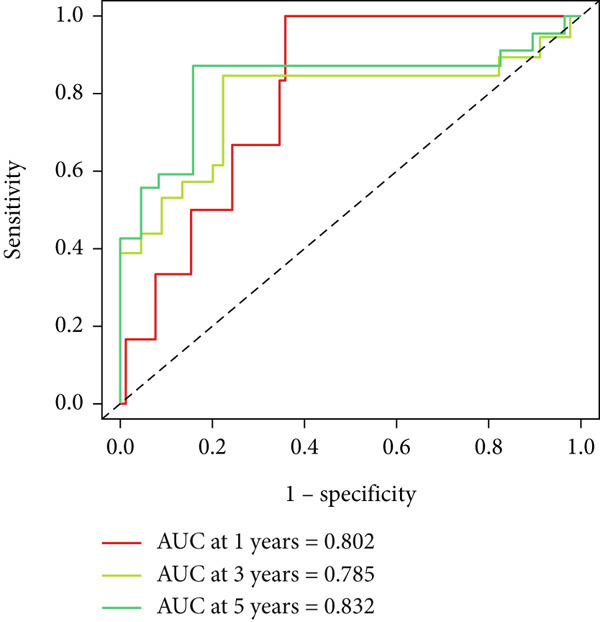
(c)

**Figure:**
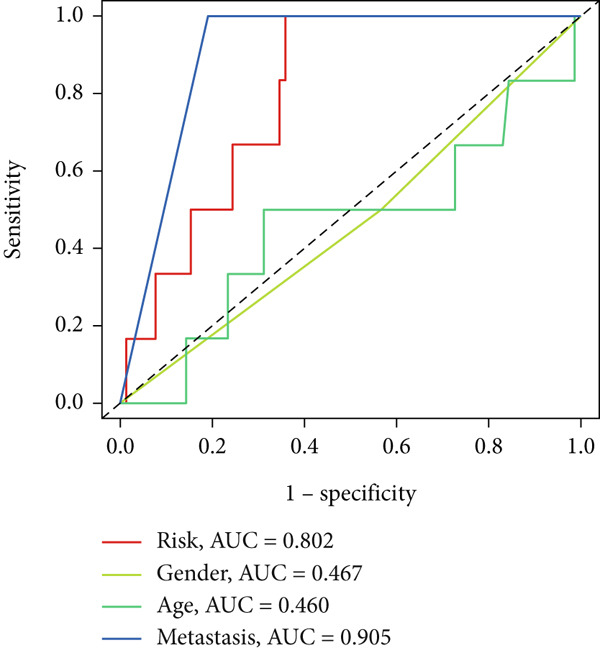
(d)

### 3.4. Enrichment Analysis of SMGs Using KEGG and GO Database

We identified 882 DEGs between the two risk groups. GO enrichment analysis revealed their involvement in keratinization, axon development, and homophilic cell adhesion via plasma membrane adhesion molecules (Figures 6a, 6b, and 6c). KEGG analysis highlighted metabolic pathways such as neuroactive ligand–receptor interaction, retinol metabolism, and porphyrin metabolism (Figures 6d, 6e, and 6f). These findings suggest that the enriched pathways may play pivotal roles in BLCA prognosis.

Figure 6Pathway enrichment analysis for high‐risk and low‐risk groups. (a–c) GO enrichment analysis for high‐risk and low‐risk groups. (d–f) KEGG pathway enrichment analysis for high‐risk and low‐risk groups.

**Figure:**
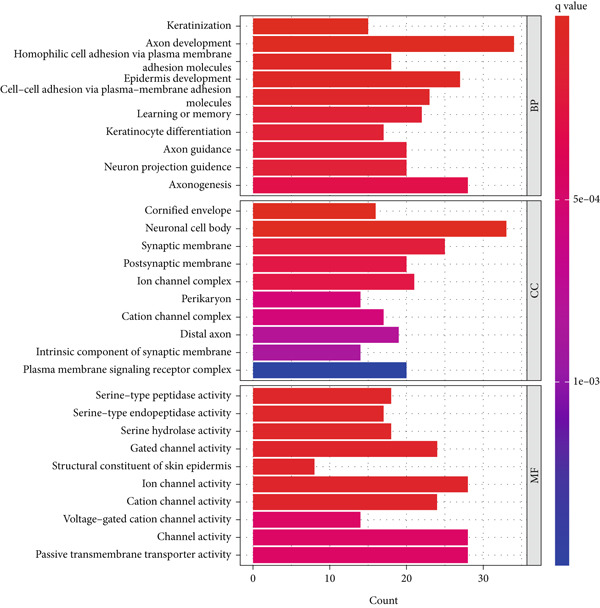
(a)

**Figure:**
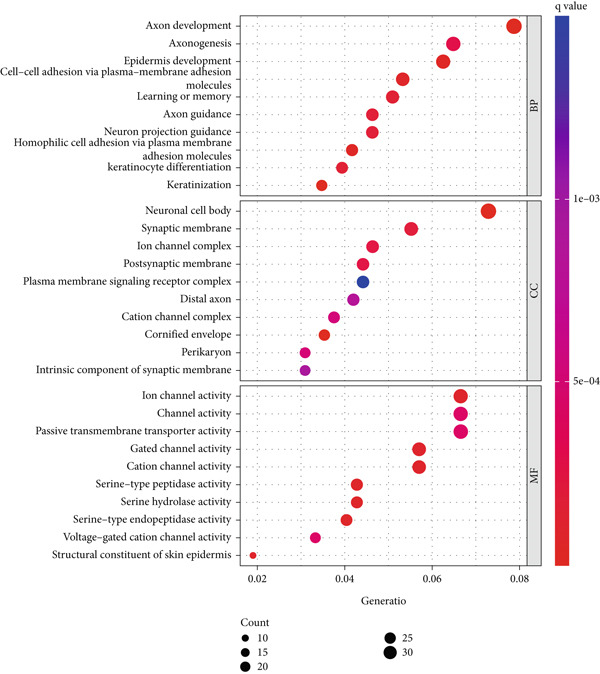
(b)

**Figure:**
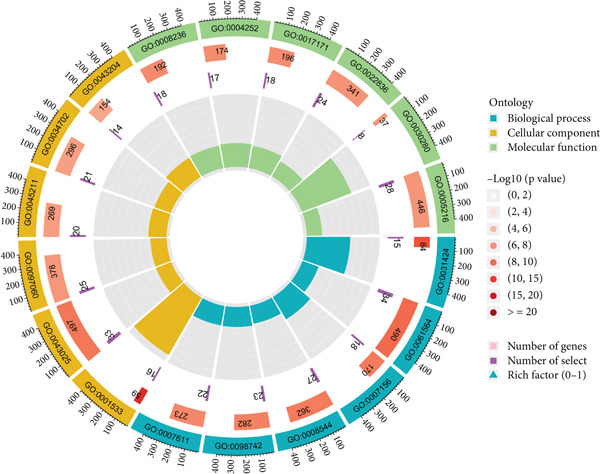
(c)

**Figure:**
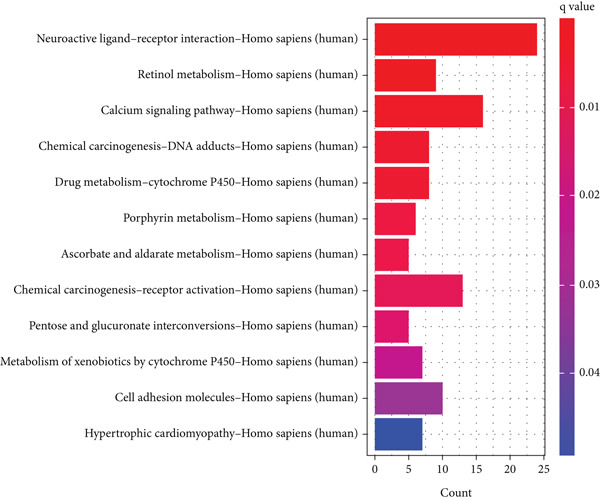
(d)

**Figure:**
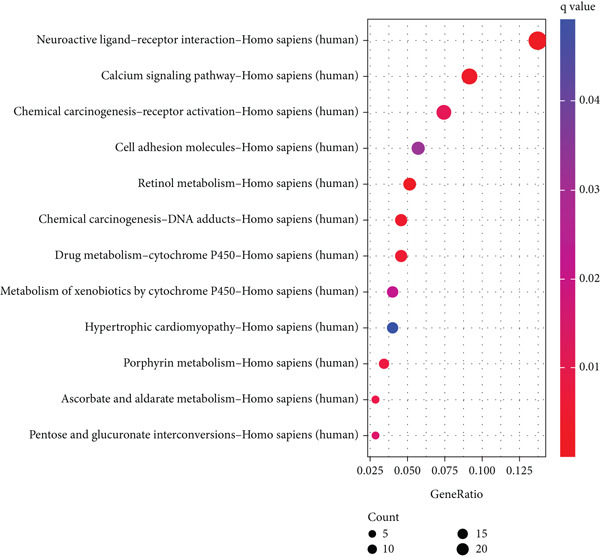
(e)

**Figure:**
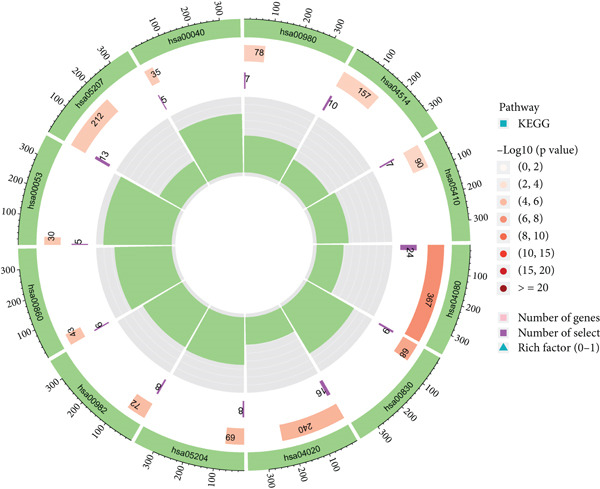
(f)

### 3.5. GSVA

GSVA revealed significant enrichment of metabolic, immune response, and cellular activity pathways in high BLCA patients (Figure 7a,b). Key differentially enriched pathways included leukocyte transendothelial migration, T‐cell receptor signaling, and lymphocyte chemotaxis regulation, suggesting distinct immune and metabolic dysregulation in high‐risk BLCA.

Figure 7GSVA analysis for high‐risk and low‐risk groups. (a) Heatmap illustrating the GO gene sets enriched in the high‐risk group compared to the low‐risk group. (b) Heatmap depicting the KEGG gene sets enriched in the high‐risk group relative to the low‐risk group.

**Figure:**
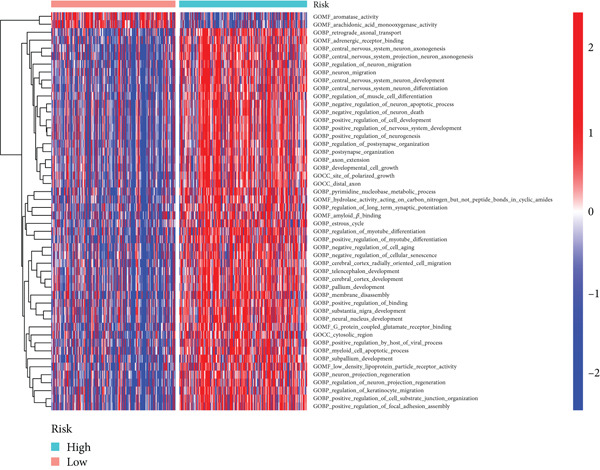
(a)

**Figure:**
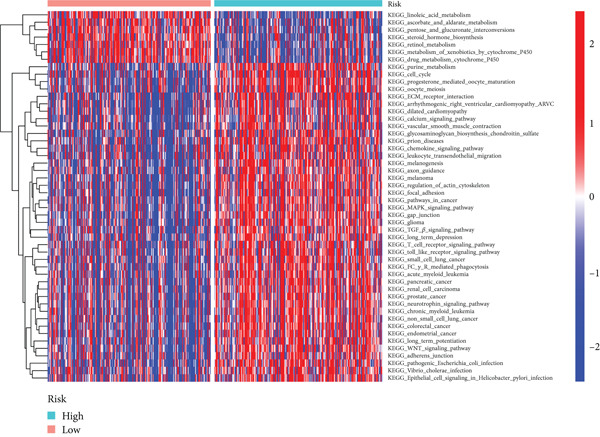
(b)

### 3.6. GSEA

GSEA identified the Top 10 enriched pathways from GO and KEGG gene sets (Figure 8a,b). Immune‐related pathways, such as adaptive immune response, immunoglobulin complex formation, antigen binding, and immunoglobulin production, were upregulated in high BLCA patients (Figures 8c, 8d, 8e, 8f, 8g, and 8h). These results further implicate immune dysfunction in BLCA prognosis.

Figure 8GSEA analysis for high‐risk and low‐risk groups. (a) Top 10 GSEA of GO gene sets. (b) Top 10 GSEA of KEGG gene sets. (c–h) Detailed GSEA of immune‐specific pathways: (c) adaptive immune response, (d) immunoglobulin complex, (e) antigen binding, (f) immunoglobulin production, (g) production of molecular mediators of immune response, and (h) T‐cell receptor complex.

**Figure:**
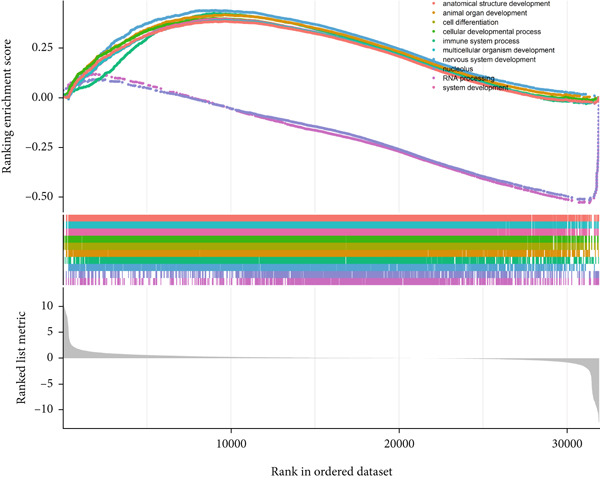
(a)

**Figure:**
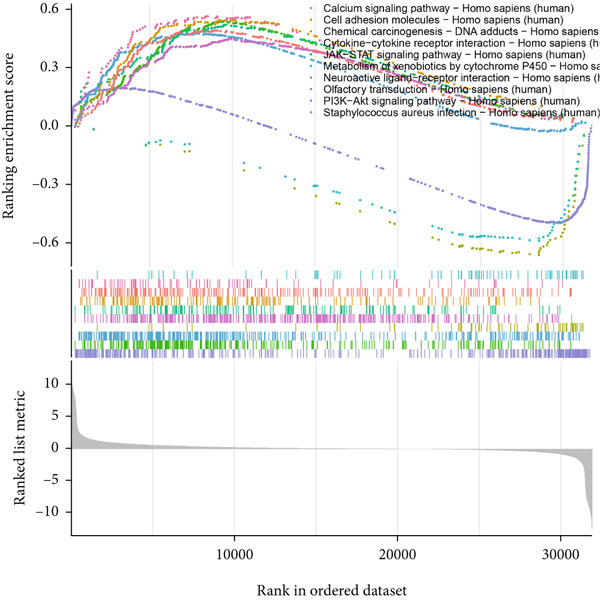
(b)

**Figure:**
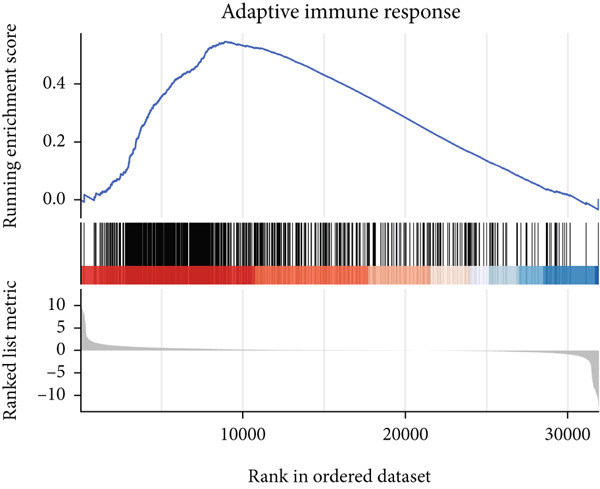
(c)

**Figure:**
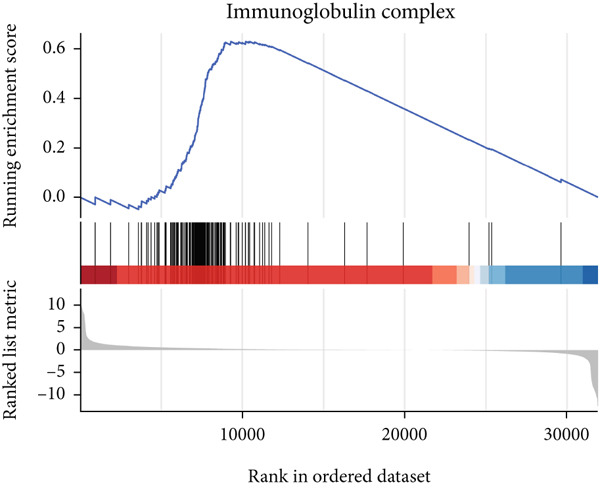
(d)

**Figure:**
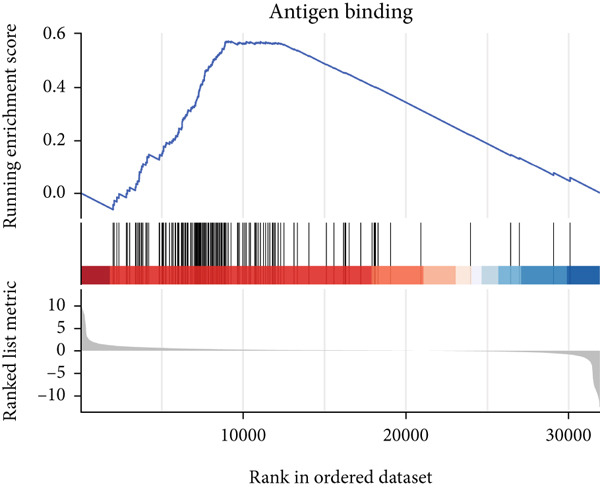
(e)

**Figure:**
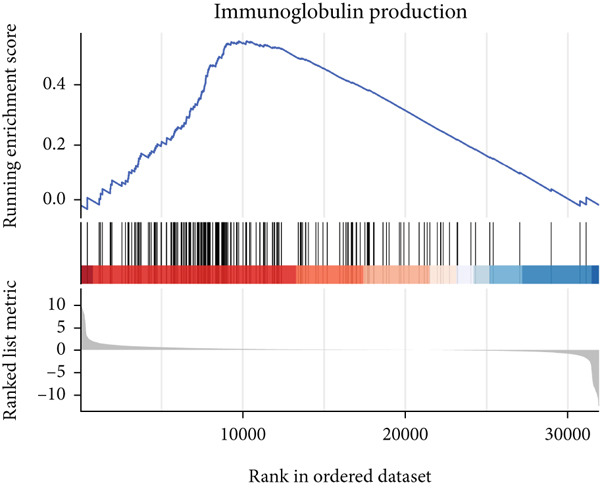
(f)

**Figure:**
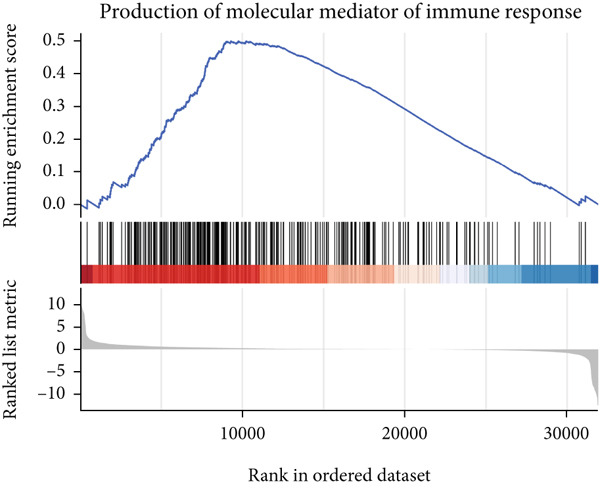
(g)

**Figure:**
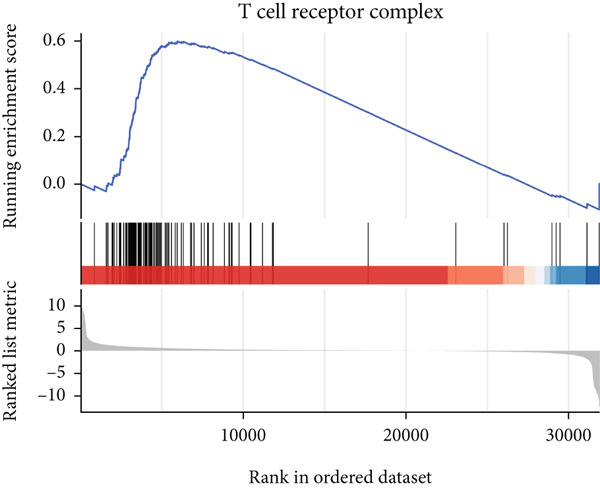
(h)

### 3.7. IME Analysis

**Figure 10:**
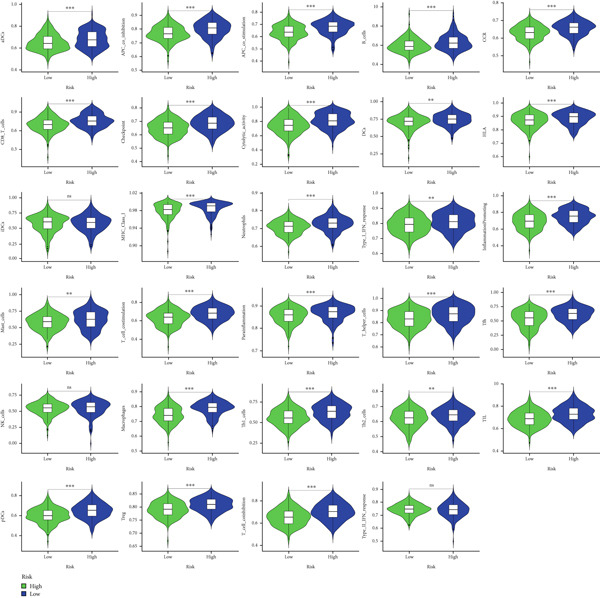
Violin plots comparing various immunological parameters between low‐risk and high‐risk groups.  ^∗^ *p* < 0.05,  ^∗∗^ *p* < 0.01, and  ^∗∗∗^ *p* < 0.001.

ESTIMATE algorithm analysis showed that high BLCA patients had elevated immune scores, stromal scores, and ESTIMATE scores, but lower tumor purity (Figures 9a, 9b, 9c, 9d, and 9e). ssGSEA confirmed higher immune cell infiltration in high BLCA patients, with 26 immune‐related functions upregulated (Figure 10). These findings suggest that immune dysfunction contributes to tumor progression and immune evasion.

Figure 9Characterization of tumor microenvironment and risk score analysis. (a) Heatmap illustrating the hierarchical clustering of tumor samples based on various immune‐related features. (b) Violin plot showing the distribution of ESTIMATEScore in low‐risk and high‐risk groups. (c) Violin plot depicting the distribution of ImmuneScore in low‐risk and high‐risk groups. (d) Violin plot illustrating the distribution of StromalScore in low‐risk and high‐risk groups. (e) Violin plot showing the distribution of TumorPurity in low‐risk and high‐risk groups.  ^∗∗∗^ *p* < 0.001.

**Figure:**
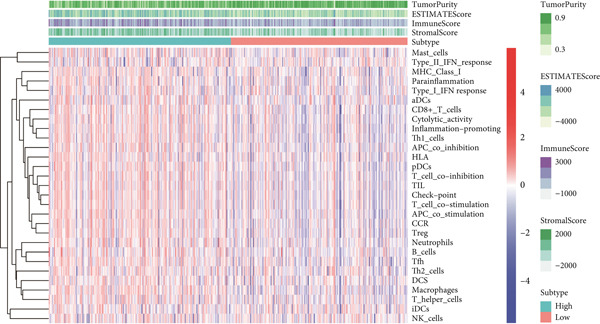
(a)

**Figure:**
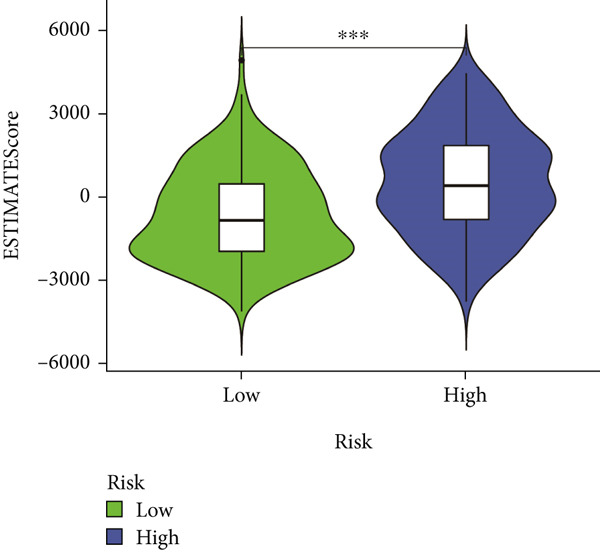
(b)

**Figure:**
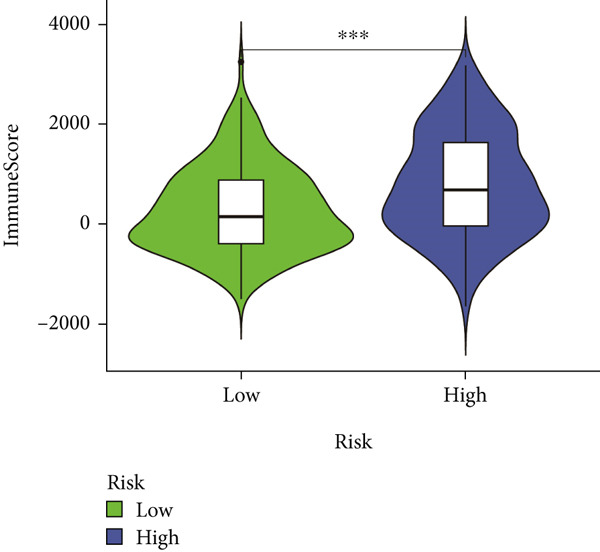
(c)

**Figure:**
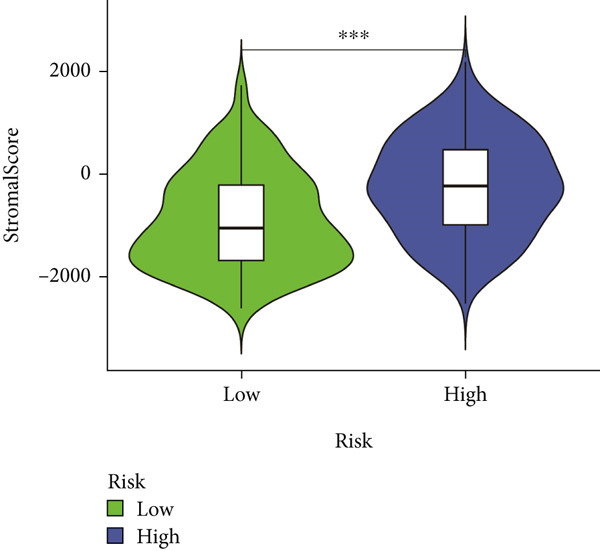
(d)

**Figure:**
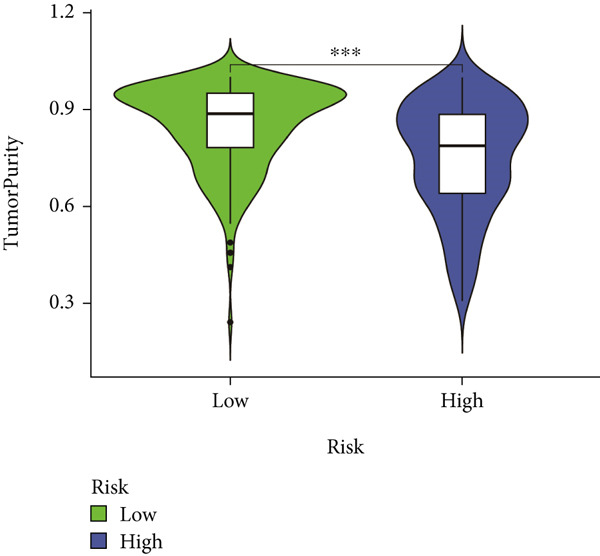
(e)

### 3.8. Single‐Cell Data Analysis

BLCA samples from the GSE database were clustered, and cell types in BLCA were annotated by the UMAP algorithm (Figure 11a). A “risk score” was calculated in the single‐cell dataset based on risk model‐included genes; its expression distribution across different cell populations was depicted on the UMAP plot (Figure 11b). The single‐cell analysis of BLCA revealed significant differences in cellular composition between high‐ and low‐risk groups. Compared to the low‐risk group, the high‐risk group exhibited a marked reduction in epithelial cell populations, accompanied by a substantial increase in fibroblast proportions (23% elevation). Concomitantly, endothelial cell fractions rose by approximately 3%, while NK/T cells demonstrated a ~1% proportional shift (Figure 11c,d). These findings underscored the critical roles of these cell types in BLCA prognosis. As illustrated in Figure 10, while NK cell–type alterations were nonsignificant in the high‐risk group, the primary immunophenotypic changes were attributed to dysregulated T‐cell expression profiles. Collectively, these observations suggest that immunocompetence alterations in the high‐risk cohort may drive epithelial–mesenchymal transition (EMT) processes, thereby elevating metastatic potential and contributing to adverse clinical outcomes through enhanced tumor progression. This mechanism highlights the importance of cellular composition shifts in shaping BLCA prognosis and therapeutic vulnerability.

Figure 11Single‐cell analysis for high‐risk and low‐risk groups in GEO database. (a) UMAP visualization of single‐cell RNA sequencing data, showing the clustering of different cell types in BLCA patients. (b) UMAP plot illustrating the distribution of high‐risk (red) and low‐risk (blue) groups across the identified cell clusters. (c) Stacked bar chart depicting the proportion of high‐risk and low‐risk groups within each cell type. (d) Comparative bar chart showing the percentage distribution of each cell type within the high‐risk and low‐risk groups.

**Figure:**
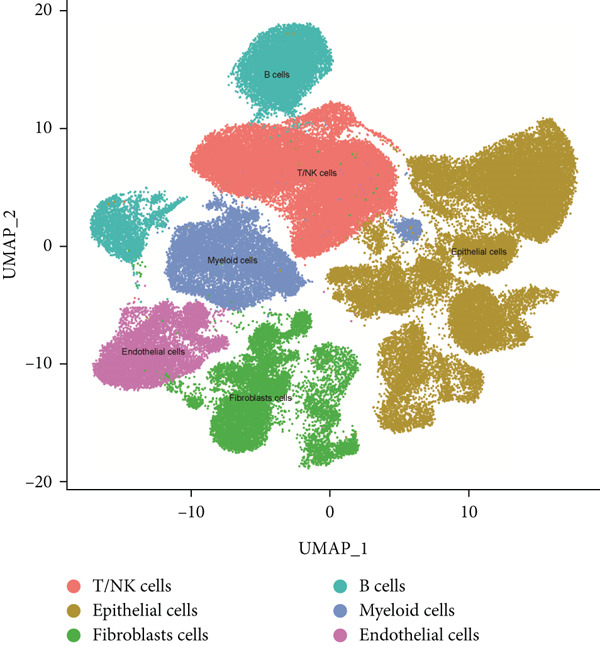
(a)

**Figure:**
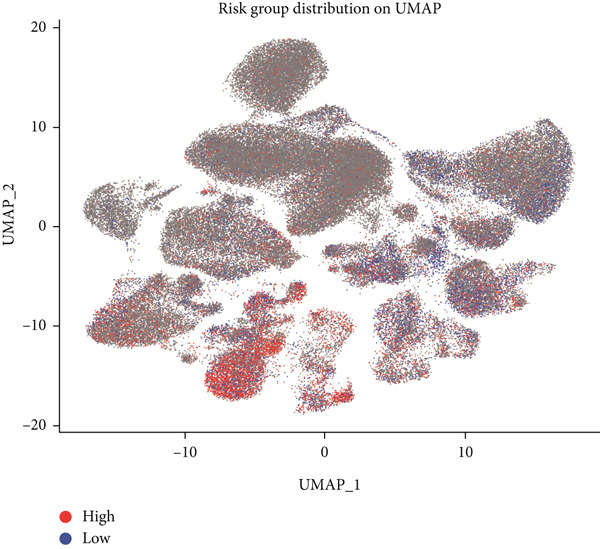
(b)

**Figure:**
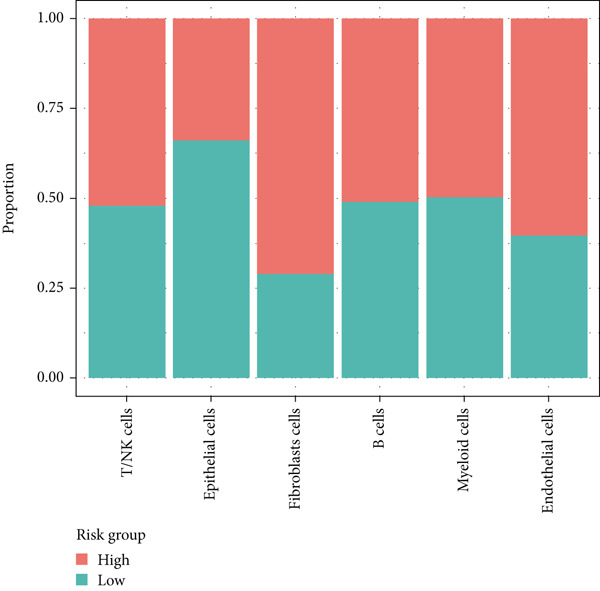
(c)

**Figure:**
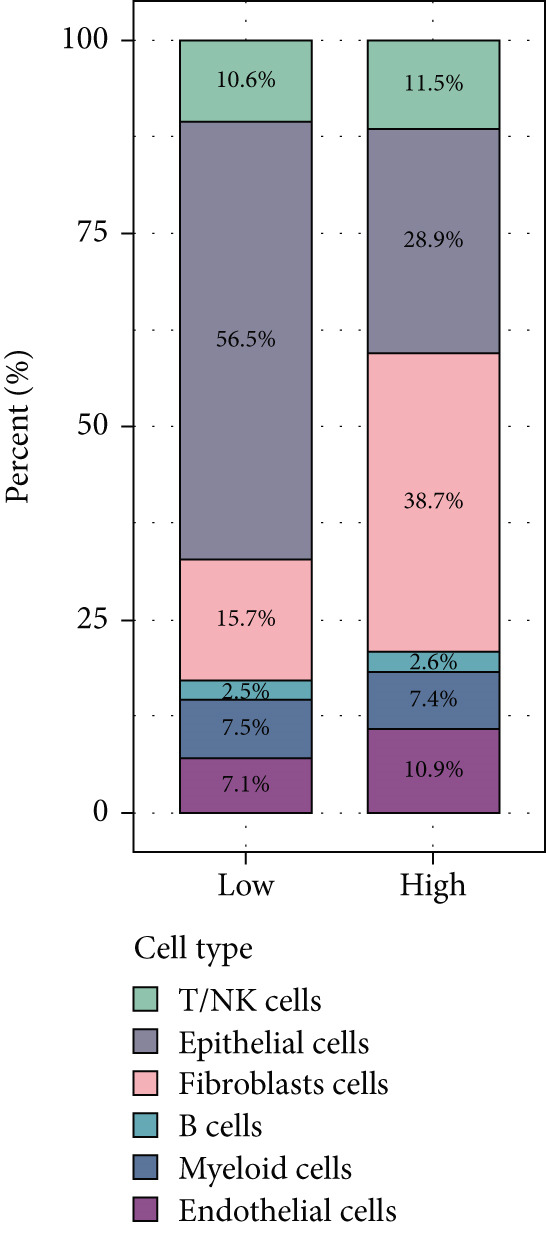
(d)

### 3.9. Immunohistochemical Validation of Key SMGs

**Figure 12:**
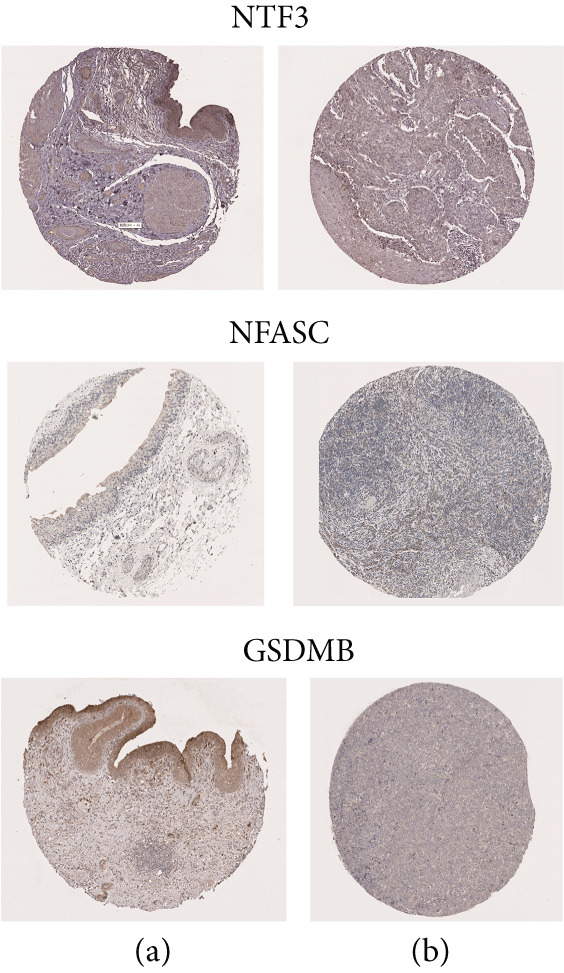
Immunohistochemical analysis of NTF3, NFASC, and GSDMB expression in (a) low‐risk and (b) high‐risk tissue sections.

Immunohistochemistry data for NTF3, NFASC, and GSDMB demonstrated differential expression between normal and BLCA tissues (Figure 12), supporting the bioinformatics findings.

### 3.10. NFASC Silencing Suppressed TCCSUP Cell Viability and Promoted Apoptosis

Following siRNA transfection into TCCSUP cells, total RNA was extracted and subjected to qRT‐PCR analysis. The results demonstrated a significant reduction in NFASC mRNA levels, confirming successful transfection and enabling subsequent experimental investigations (Figure 13a).

Figure 13Effects of NFASC knockdown on mRNA expression, TCCSUP cell proliferation, and apoptosis. (a) Relative mRNA levels of the target gene in control, si‐NC, and si‐NFASC groups. (b) EDU/DAPI ratio in control, si‐NC, and si‐NFASC groups, indicating cell proliferation activity. (c) Immunofluorescence staining for EDU (red) and DAPI (blue) in control, si‐NC, and si‐NFASC groups. (d) Flow cytometry analysis of apoptosis in control, si‐NC, and si‐NFASC groups.  ^∗∗^ *p* < 0.01;  ^∗∗∗∗^ *p* < 0.0001.

**Figure:**
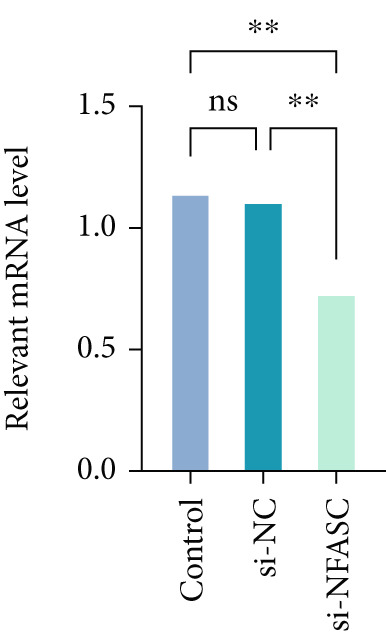
(a)

**Figure:**
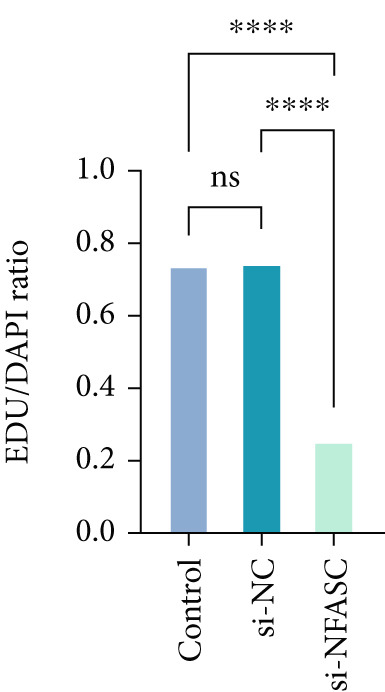
(b)

**Figure:**
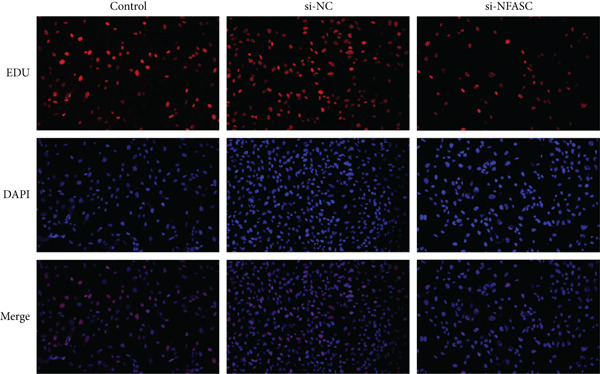
(c)

**Figure:**
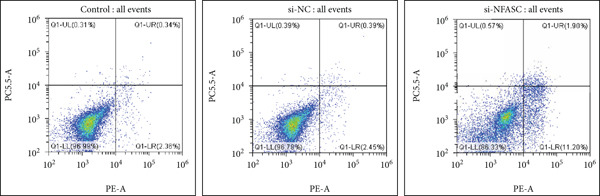
(d)

Cell proliferation and viability were assessed using an EDU assay. Fluorescently labeled active substances incorporated into newly synthesized DNA molecules resulted in red‐positive cells, while DAPI staining highlighted nuclei in blue. The experimental data revealed a marked decrease in the proportion of EDU‐positive cells upon NFASC silencing, indicating suppressed proliferation and viability (Figure 13b,c). These findings further validated NFASC as a risk gene in BLCA.

Flow cytometry analysis revealed a significant increase in the apoptosis rate following NFASC silencing (Figure 13d). This underscored the critical role of NFASC in promoting the survival and proliferation of TCCSUP cells.

### 3.11. NFASC Silencing Attenuated Migration and Invasion in TCCSUP Cells

Tumor cell migration and invasion are pivotal mechanisms underlying cancer metastasis. To evaluate the impact of NFASC on these processes, wound healing assays, Transwell assays, and colony formation assays were performed. The wound healing assay demonstrated a significantly slower healing rate in the si‐NFASC group compared to the control and si‐NC groups under identical conditions (Figure 14a,b). Similarly, the Transwell assay revealed a substantial reduction in the number of migrating cells upon NFASC silencing (Figure 14c,d). The colony formation assay further supported these observations, with the si‐NFASC group exhibiting fewer colonies than the control groups (Figure 14e). Collectively, these results indicated that NFASC silencing effectively inhibits the migratory and invasive capabilities of TCCSUP cells, corroborating our predictive model and highlighting its potential as a therapeutic target.

Figure 14Effects of NFASC knockdown on TCCSUP cell migration and proliferation. (a) Representative images of wound healing assays in control, si‐NC, and si‐NFASC cells at 0, 24, and 48 h postscratch. (b) Quantification of wound healing percentage at 24 h (left) and 48 h (right) postscratch. (c) Cell count analysis in Transwell assay. (d) Representative images of crystal violet staining for cell migration in control, si‐NC, and si‐NFASC cells. (e) Representative images of colony formation assays in control, si‐NC, and si‐NFASC cells.  ^∗∗^ *p* < 0.01;  ^∗∗∗∗^ *p* < 0.0001.

**Figure:**
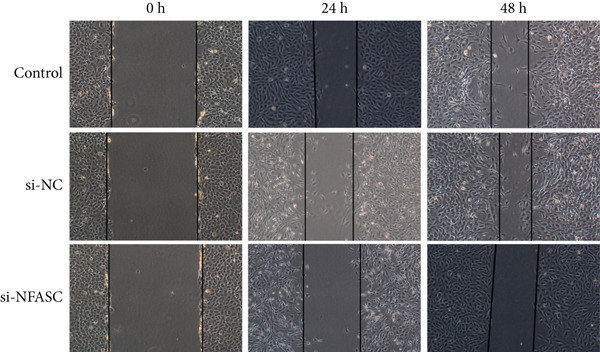
(a)

**Figure:**
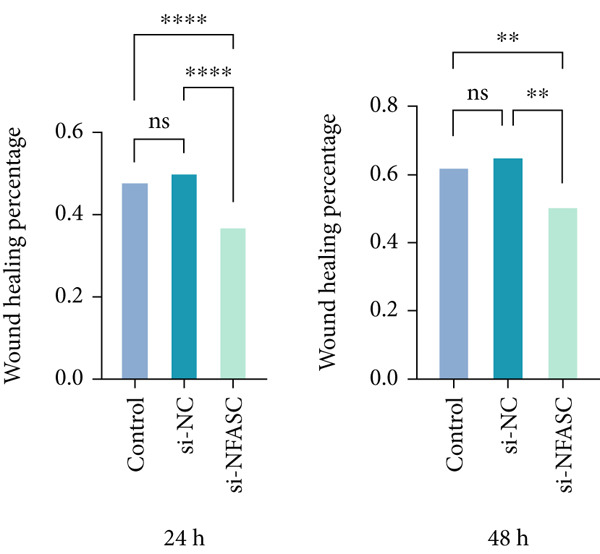
(b)

**Figure:**
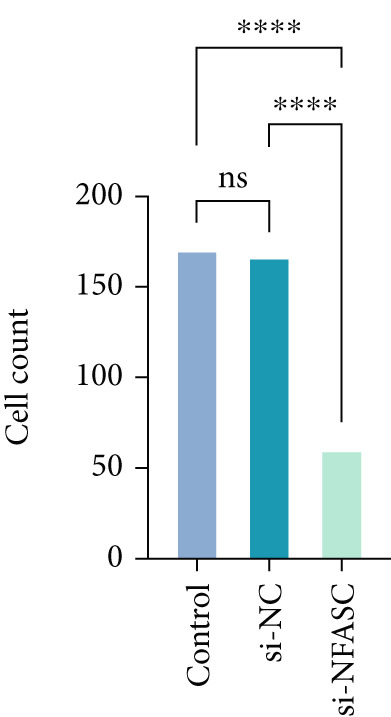
(c)

**Figure:**
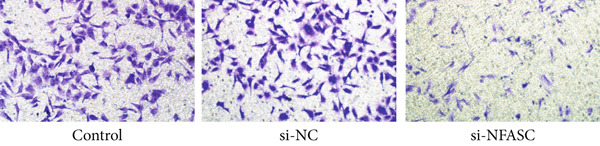
(d)

**Figure:**
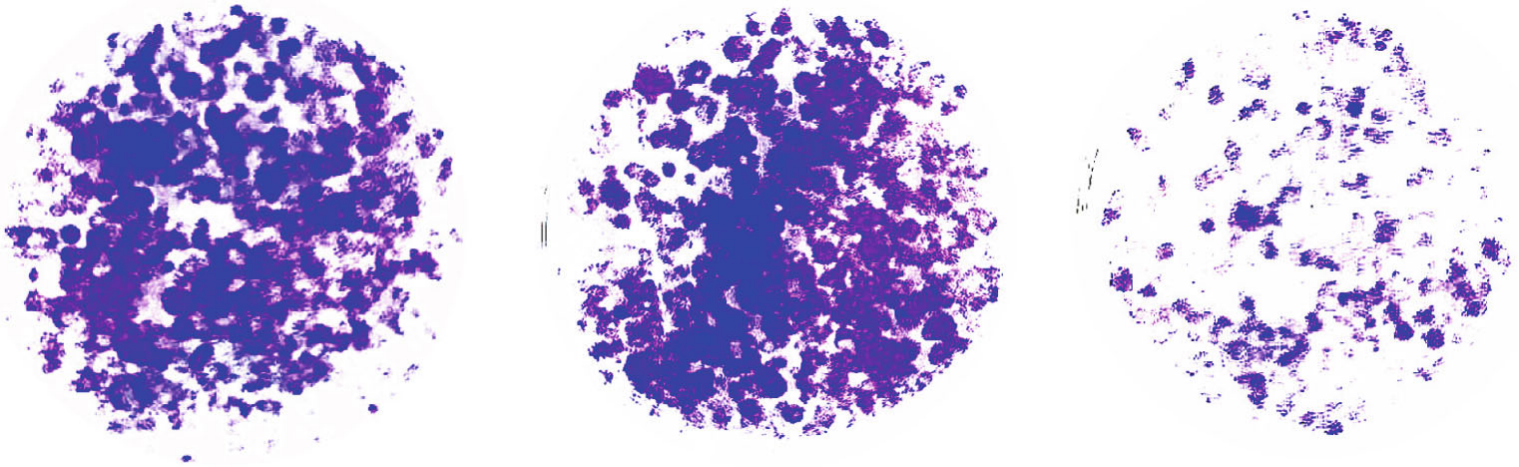
(e)

## 4. Discussion

Metabolic reprogramming (MRG) is a hallmark of cancer, with each metabolic state exhibiting a distinct genetic signature that influences disease progression [17]. Alterations in tumor stroma metabolism, particularly glucose MRG, play a significant role in oncogenic pathways [18–20]. The Myc protein, a key metabolic regulator, drives MRG by modulating glucose and glutamine metabolism, as well as serine production, thereby fueling cancer cell growth [20].

Despite advancements in BLCA diagnosis and treatment over the past two decades, high relapse rates and the risk of progression to invasive disease render it one of the most costly cancers to manage [21]. Challenges such as recurrence, antimicrobial resistance, and rapid disease progression underscore the urgent need for novel biomarkers to improve clinical detection and therapeutic strategies [22].

In this study, we pioneered the exploration of the relationship between BLCA and SMGs. Using LASSO Cox and UniCox regression analyses, we developed a predictive BLCA risk score model based on 54 DEGs from TCGA datasets. The model effectively stratified patients into low and high BLCA score groups with distinct survival outcomes, a finding validated in both training and test cohorts. MultCox analysis further confirmed the model′s independence as a prognostic factor.

Immunological profiling revealed that high BLCA score patients exhibited elevated levels of immune‐inflamed and immunosuppressive cells, including Tregs, APC costimulation, and checkpoint molecules, suggesting their potential candidacy for immunotherapy. The ESTIMATE algorithm was employed to assess tumor purity and immune infiltration, revealing that patients with favorable prognoses had lower tumor purity and higher immune scores. Subsequent ssGSEA analysis corroborated these findings, demonstrating immune dysregulation in high BLCA patients, characterized by elevated activity in 26 of 29 immune‐related pathways. This immune dysfunction likely contributes to poorer prognoses.

Our study highlights the role of SM dysregulation in TIME anomalies, which adversely affect BLCA outcomes [23]. Sphingolipids, particularly gangliosides like GD3 and GD2, promote tumorigenesis, whereas monosialyl gangliosides (e.g., GM1 and GM3) exhibit tumor‐suppressive properties [24, 25]. These lipids modulate cellular signaling via *cis*‐ and *trans*‐binding interactions, influencing pathways involving growth factors, integrins, and immune cell activity [26]. For instance, in melanomas, AKT, p130Cas, and paxillin drive carcinogenesis and migration, potentially mediated by Src family proteins [27].

Among the genes of interest (NTF3, NFASC, and GSDMB), NFASC emerged as a high‐risk gene in BLCA. In vitro experiments using TCCSUP cells demonstrated that NFASC silencing inhibited proliferation, induced apoptosis, and suppressed migration and invasion, aligning with our bioinformatics predictions. These findings underscore the reliability of our model and its potential for risk stratification and targeted therapy development.

In recent years, the therapeutic landscape of BLCA has undergone a significant transformation with the emergence of immune checkpoint inhibitors (ICIs), targeted therapies, and especially antibody‐drug conjugates (ADCs). Agents such as enfortumab vedotin, sacituzumab govitecan, and disitamab vedotin (RC48‐ADC) have demonstrated promising efficacy in patients with advanced or metastatic BLCA, particularly those who are resistant to chemotherapy or immunotherapy [28]. Real‐world studies have shown that neoadjuvant chemoimmunotherapy (e.g., tislelizumab combined with gemcitabine and cisplatin) achieves significantly higher pathological complete response (pCR) and downstaging rates compared to chemotherapy or immunotherapy alone. Furthermore, RC48‐ADC combined with ICIs has achieved a pathological response rate of 75.5% and a 1‐year disease‐free survival rate of 97.4% in patients with HER2‐positive MIBC [29]. These findings not only highlight the clinical potential of ADC‐based combination regimens but also underscore the urgent need for predictive models to guide treatment selection.

Our risk score model, developed based on SMGs, reflects key immune characteristics of the TME, including both immune activation and suppression. Given the role of sphingolipids in regulating membrane architecture, receptor clustering, and signal transduction, this model may also offer predictive value for response to ADC monotherapy or ADC‐ICI combination regimens. Moreover, high‐risk genes identified in our model, such as NFASC, may influence tumor behavior and treatment sensitivity by modulating the TME. Future studies integrating our model with drug‐specific biomarkers such as HER2 and HSPA1A may enable more accurate identification of patients likely to benefit from ADC‐based therapies, thereby contributing to precision medicine in BLCA.

### 4.1. Limitations

There are several limitations to this study. Due to funding constraints and limited availability of clinical samples, our experimental validation relied on publicly available immunohistochemistry data. Although these data partially support our findings, future studies should involve more patient‐derived tissue samples and functional experiments to elucidate the mechanistic roles of SMGs in BLCA progression.

In addition, while our study revealed a correlation between SMG dysregulation and immune microenvironment remodeling, it lacks direct causal evidence. Functional in vivo or in vitro experiments—such as gene knockout or overexpression of key SMGs—are needed to determine whether sphingolipid metabolites directly modulate immune cell function or whether immune modulation influences sphingolipid signaling in return.

Furthermore, our prognostic model was constructed using core clinical variables such as age, gender, and tumor stage. However, important real‐world factors, including lifestyle characteristics (e.g., smoking history), comorbidities (e.g., diabetes and hypertension), and treatment history (e.g., chemotherapy or immunotherapy), were not incorporated. These variables may affect patient prognosis and introduce confounding bias when applying the model in clinical settings. Future studies should aim to optimize the model by integrating a broader spectrum of clinical and biological variables to improve its predictive accuracy and clinical applicability.

## 5. Conclusion

This study establishes a correlation between SM and BLCA, enabling stratification of patients into high‐ and low‐risk groups. Immune and functional analyses revealed that sphingolipid dysregulation impairs immune status, exacerbating disease prognosis. These insights provide a foundation for personalized risk assessment and the development of targeted therapies tailored to individual patient profiles.

## Ethics Statement

The authors have nothing to report.

## Consent

The authors have nothing to report.

## Disclosure

This manuscript has been presented as a preprint in Research Square.

## Conflicts of Interest

The authors declare no conflicts of interest.

## Author Contributions

Tianshi Wu and Ruipeng Jia: conceptualization and visualization; Zechun Peng and Jie Yang: original manuscript writing; Zechun Peng and Jie Yang: formal analysis; Tianshi Wu: data curation; Tianshi Wu, Ruipeng Jia, Jie Yang, and Zechun Peng: writing—review and editing and project administration. Zechun Peng and Jie Yang contributed equally to this work.

## Funding

This study was funded by the Hainan Provincial Natural Science Foundation of China (ZDYF2024SHFZ122, 820MS142, and 822QN472) and the Hainan Province Clinical Medical Center.

## Data Availability

The study provides access to the datasets through online repositories. You can find the repository/repositories′ names and corresponding accession number(s) in the following link: https://xenabrowser.net/datapages/.
